# Constipation

**Published:** 1863-06

**Authors:** 


### CONSTIPATION.

Dr. William Bodenhamer, of New-York, has been more successful in treating those conditions of the lower bowel which require surgical aid than any other physician at home or abroad. The following, taken from his last-published work on those maladies, merits universal practical personal attention:

"Continued care and anxiety of mind are also causes of constipation of the bowels. The great anxiety to which those are subjected who are continually and actively engaged in their business or profession, exerts a powerful influence upon the functions of the alimentary canal; it not only depresses the nervous system, and causes indigestion, but also renders torpid the peristaltic action of the bowels. As a confirmation of the truth of this, as soon as such persons emancipate themselves from the cares and anxieties of their business, by taking an excursion into the country, the mind soon recovers its wonted cheerfulness, the spirits their elasticity, and the bowels their normal function. Indeed, when we take into consideration the contentions, the competitions, and the responsibilities which this class of persons have daily to encounter, especially in large cities, it is a matter of no surprise that they should suffer from languor and depression, from indigestion and from constipation, with all its numerous evil consequences. To be forcibly impressed with the extent of the evils resulting from this cause, it is only necessary to pass along Broadway or the Bowery early in the morning, or late in the evening, and behold the multitude of anxious and careworn countenances which one sees daily at those hours in these two great thoroughfares of New-York. This spectacle will furnish a good commentary upon the *wear* and *tear* of human life.

"*Sedentary habits*, or the sedentary occupations of life, greatly tend to constipation and inactivity of the bowels — hence men of literary pursuits, or those closely occupied in study, as students, or employed at the desk or counter, as clerks and accountants; or those who are confined to seats, as tailors, seamstresses, milliners, etc., are all extremely liable to suffer greatly from confined bowels. The want of bodily exercise generally lessens the demand for food, weakens the digestive organs, and indigestion and constipation are almost a necessary consequence. Witness the large number of poor young women in New-York who sit and ply the needle from daylight in the morning till ten or eleven o'clock at night, with scarcely intermission enough to take their meals, or to attend to the calls of nature. This employment, so unremitting in exercise, continues from Monday morning to Saturday night '*in one wearyiny, unvarying sedentary position*.' No class of persons in this metropolis are so deserving of sympathy and commiseration as these poor women. Spirit of Tom Hood, look down upon these '*white slaves*' and pity them. Well didst thou sing:

"'O men with children dear!
O men with sisters and wives!
It is not *linen* you're wearing out,
But human creatures' lives!
Stitch, stitch, stitch,
In poverty, hunger and dirt,
And still she sews with a double thread
A shroud as well as a shirt.'

"How many sing that '*song of the shirt*' in New-York today whilst they sew their shrouds?

"Some of the causes of constipation among the richer and higher classes of people are the modern and irregular hours of society, including late breakfasts, late dinners, and all the long nocturnal pastimes of music and dancing in crowded and heated rooms, etc.; all of which necessarily imply many contraventions and restraints of the laws and of the regular discharge of the functions of nature."

